# Clinical and Diagnostic Features of Post-Acute COVID-19 Vaccination Syndrome (PACVS)

**DOI:** 10.3390/vaccines12070790

**Published:** 2024-07-18

**Authors:** Anna Katharina Mundorf, Amelie Semmler, Harald Heidecke, Matthias Schott, Falk Steffen, Stefan Bittner, Karl J. Lackner, Karin Schulze-Bosse, Marc Pawlitzki, Sven Guenther Meuth, Frank Klawonn, Jana Ruhrländer, Fritz Boege

**Affiliations:** 1 Central Institute for Clinical Chemistry and Laboratory Diagnostics, Medical Faculty, University Hospital Düsseldorf, Heinrich-Heine-University, 40225 Düsseldorf, Germany; annakatharina.mundorf@med.uni-duesseldorf.de (A.K.M.); ansem100@uni-duesseldorf.de (A.S.); karin.schulze-bosse@med.uni-duesseldorf.de (K.S.-B.); 2 CellTrend GmbH, 14943 Luckenwalde, Germany; heidecke@celltrend.de; 3 Division for Specific Endocrinology, Medical Faculty, University Hospital Düsseldorf, Heinrich-Heine-University, 40225 Düsseldorf, Germany; matthias.schott@med.uni-duesseldorf.de; 4 Department of Neurology, Focus Program Translational Neuroscience (FTN) and Immunotherapy (FZI), Rhine Main Neuroscience Network (RMN2), Medical Center, Johannes Gutenberg University Mainz, 55131 Mainz, Germany; falk.steffen@unimedizin-mainz.de (F.S.); bittner@uni-mainz.de (S.B.); 5 University Medical Center, Johannes Gutenberg University Mainz, 55122 Mainz, Germany; karl.lackner@unimedizin-mainz.de; 6 Department of Neurology, Medical Faculty, Heinrich-Heine-University, 40225 Düsseldorf, Germany; marcguenter.pawlitzki@med.uni-duesseldorf.de (M.P.); svenguenther.meuth@med.uni-duesseldorf.de (S.G.M.); 7 Biostatistics Research Group, Helmholtz Centre for Infection Research, 38124 Braunschweig, Germany; frank.klawonn@helmholtz-hzi.de; 8 Department of Computer Science, Ostfalia University, 38302 Wolfenbüttel, Germany; 9 Selbsthilfegruppe Post-Vac-Syndrom Deutschland e.V., 34121 Kassel, Germany; jana.ruhrlaender@gmx.de

**Keywords:** post-acute COVID-19 vaccination syndrome, PACVS, interleukin-6, interleukin-8, malaise/chronic fatigue, cognitive impairment, peripheral nerve dysfunction

## Abstract

Post-acute COVID-19 vaccination syndrome (PACVS) is a chronic disease triggered by SARS-CoV-2 vaccination (estimated prevalence 0.02%). PACVS is discriminated from the normal post-vaccination state by altered receptor antibodies, most notably angiotensin II type 1 and alpha-2B adrenergic receptor antibodies. Here, we investigate the clinical phenotype using a study registry encompassing 191 PACVS-affected persons (159 females/32 males; median ages: 39/42 years). Unbiased clustering (modified Jaccard index) of reported symptoms revealed a prevalent cross-cohort symptomatology of malaise and chronic fatigue (>80% of cases). Overlapping clusters of (i) peripheral nerve dysfunction, dysesthesia, motor weakness, pain, and vasomotor dysfunction; (ii) cardiovascular impairment; and (iii) cognitive impairment, headache, and visual and acoustic dysfunctions were also frequently represented. Notable abnormalities of standard serum markers encompassing increased interleukins 6 and 8 (>80%), low free tri-iodine thyroxine (>80%), IgG subclass imbalances (>50%), impaired iron storage (>50%), and increased soluble neurofilament light chains (>30%) were not associated with specific symptoms. Based on these data, 131/191 participants fit myalgic encephalomyelitis/chronic fatigue syndrome (ME/CFS) and simultaneously also several other established dysautonomia syndromes. Furthermore, 31/191 participants fit none of these syndromes. In conclusion, PACVS could either be an outlier of ME/CFS or a dysautonomia syndrome sui generis.

## 1. Introduction

In rare cases, SARS-CoV-2 mRNA vaccination entails a variety of chronic sequelae summarily addressed as post-acute COVID-19 vaccination syndrome (PACVS) [1]. PACVS has an estimated prevalence of 0.02% and imposes as a distinct etiology [1,2]. However, it shares features with complex multisystemic dysautonomia syndromes not related to vaccination, such as myalgic encephalomyelitis/chronic fatigue syndrome (ME/CFS) [3,4], postural tachycardia syndrome (POTS) [5], fibromyalgia/chronic pain syndrome [6], small fiber neuropathy (SFN) [7], and mast cell activation syndrome (MCAS) [8]. Similarly, it shares features with prolonged courses and chronic sequelae of COVID-19 (long COVID or post-acute COVID-19 syndrome, PACS [1]), which is thought to be due to common pathogenic mechanisms such as spike S1 protein persistence [9,10].

Initial results of our recent study on 191 carefully selected participants [2] suggest that the serological vaccination response of PACVS-affected individuals is significantly altered. Thus, PACVS could be objectively discriminated from the normal post-vaccination state by diagnostic blood markers, most notably increases in interleukins 6 and 8 and alterations in certain receptor antibodies. While these alterations of blood markers are not disease-specific, they lend support to the notion that PACVS is a somatic syndrome. However, the clinical presentation of this putative syndrome is so far poorly defined.

Here, we attempt to more precisely delineate the disease phenotype of PACVS by (i) evaluating a host of clinical data registered in the context of the above study [2] and (ii) analyzing a broad panel of established blood markers indicative of specific etiologies and organ dysfunctions. Based on these data, we propose a consolidated clinical phenotype of PACVS, which may enable a deeper comprehension of the fundamental pathological mechanism of this novel syndrome and hopefully promote the development of dedicated therapeutic concepts.

## 2. Materials and Methods

### 2.1. Study Participants

Recruitment and selection of study participants has previously been described [2]. Briefly, *n* = 159 females and *n* = 32 males (mean/median age = 40/39 years) suffering from chronic sequelae of SARS-CoV-2 mRNA vaccination for five months or more were included [2]. Clinical trial protocols were approved by the local ethics board of Heinrich-Heine University Düsseldorf (study number 2022-1948). The investigation conforms to the principles outlined in the World’s Medical Association Declaration of Helsinki. Before inclusion, all participants gave written informed consent.

### 2.2. Laboratory Measurements

PACVS-associated features of blood markers were determined in blood samples obtained from the study participants by cubital vein puncture. Blood samples were kept at 4 °C until delivery to the laboratory within 48 h. Serum was prepared immediately upon receipt and stored at −20 °C or −80 °C until analysis. Hemolytic sera were rejected, based on enhanced values of free hemoglobin. Receptor antibodies were determined with commercially available immunoassays (CellTrend GmbH, Luckenwalde, Germany) [2]. SARS-CoV-2 serology was monitored by panIg reactivity against SARS-CoV-2 spike S1 protein (SAB) and SARS-CoV-2 nucleocapsid protein (NAB) [11]. Serum neurofilament light chains (sNFL) were measured using the SIMOA NF-light V2 Advantage Kit (Quanterix Corporation, Billercia, MA 01821, USA). sNFL values were adjusted to body mass index (BMI) and age, and z-scores were calculated according to a published control cohort [12]. All other laboratory tests were performed by accredited routine laboratory diagnostic procedures and related to established routine reference values. Reference values for total IL-8 were derived from reference values of free IL-8 (<62 pg/mL) by multiplying by five, estimating the erythrocyte-bound fraction at 80%, according to [13].

### 2.3. Clinical Data

Clinical data were retrieved from an online registry of symptoms established for ME/CSF, POTS, MCAS, and SFN [4,5,7,8] and/or those frequently exhibited by subjects suffering from chronic post-vaccination sequelae (Table 1). The maximal number of queries in the questionnaire was limited by ethical concerns. Therefore, not all guideline criteria of ME/CSF, POTS, MCAS, or SFN [4,5,7,8] could be registered. Incidentally, a very similar selection of symptoms was previously monitored in a survey of COVID-19 sequelae [14]. Of note, symptoms registered for the study subjects (i) had been triggered by SARS-CoV-2 mRNA vaccination and (ii) had persisted at the time of sample acquisition for five months or more. Symptoms were registered based on self-assessment, which in most cases was corroborated by external physicians. However, medical case histories were not subjected to systematic analysis by the study team, nor were participants subjected to direct physical examination by the study team.

### 2.4. Statistical Methods

Graph Pad Prism 9 for Windows or Apple Macintosh (released 2020) was used for quantitative data analysis. Non-normally distributed data (Shapiro–Wilk) were descriptively analyzed by mean/median values and interquartile ranges. Differences between study subjects and controls were analyzed by the Mann–Whitney U test (two-tailed). Spearman’s correlations were based on 95% confidence limits and assumed to be good at r ≥ 0.7. Statistical significance was assumed at *p* < 0.0001. Missing data was handled by listwise deletion. Participants were assigned to ME/CSF, POTS, MCAS, or SFN when reporting more than the cohort average of related symptoms as specified in published guidelines [4,5,7,8] and/or when having been explicitly diagnosed with that syndrome by a physician. Overlap of syndrome assignments was analyzed by Chi-square tests. Draw-io V21.6.1 (JGraph Ltd., Northampton, UK, release 2023) was used for extended Venn diagram analysis.

Hierarchical clustering of symptoms was performed using a modified Jaccard index as a similarity measure, defined as the mean of the standard Jaccard index and the reversed Jaccard index in which events and non-events are exchanged. The modified Jaccard index was used because some symptoms occurred in the majority of patients. Clusters were selected at the level h = 1.8 using the cut-tree function of the computer language R, based on medical knowledge. The patients were then assigned to the eight clusters previously defined using the modified Jaccard index. This was performed in three different ways: the proportion of symptoms in the cluster (prop), the Jaccard index with the symptoms in the cluster, and the modified Jaccard index (average of the Jaccard index and the Jaccard index of the negated values).

The association of blood marker abnormalities (ferritin index, fT3, IgG3, IgG4, and z-score of sNFL) with symptom clusters was tested by plotting the scatter of each blood marker exhibiting abnormalities against corresponding cluster assignments of the patients based on (i) the proportion of cluster-specific symptoms presented by the patient, (ii) the Jaccard index, or (iii) a modified Jaccard index defined as the mean of the classic Jaccard index and the reversed Jaccard index with respect to the symptoms not indicated by the corresponding patient and not lying within the symptom cluster.

## 3. Results

### 3.1. Prevalence of ME/CFS-, POTS-, SFN- and MCAS-Associated Symptoms in PACVS

**Figure 1:**
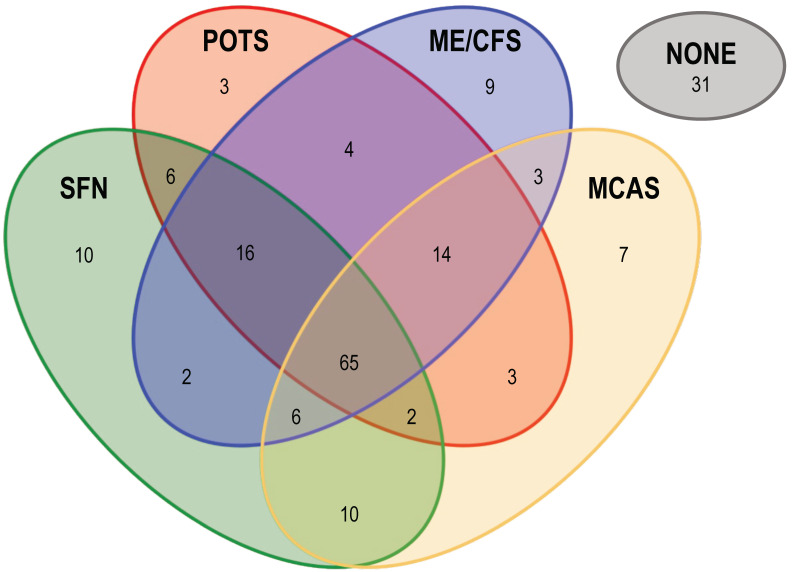
Overlap of complex dysautonomia syndromes with symptoms reported by the PACVS study cohort. ME/CSF, POTS, MCAS, and SFN were assigned to the study participants according to published guidelines [4,5,7,8]. PACVS subjects were assigned a given syndrome if exhibiting *n* ≥ the cohort average of symptoms associated with that syndrome or if diagnosed after vaccination with that syndrome by a physician. A total of *n* = 31 PACVS subjects could not be assigned to any of the four syndromes (labeled “NONE”). Numbers indicate the *n* of study participants assigned to a given syndrome or syndrome intersection.

Symptoms registered for PACVS subjects participating in this study are listed in Table 1. Symptoms conforming to criteria established for ME/CSF, POTS, MCAS, or SFN [4,5,7,8] were the ones most frequently registered. Based thereon, a majority of PACVS cases in the study could be assigned to at least one of these syndromes (Figure 1). However, study participants also reported a variety of disease symptoms not associated with ME/CSF, POTS, MCAS, or SFN (Table 1). Covariance analysis of syndrome assignments showed that only 29/191 PACVS cases in the study could be unambiguously assigned to either ME/CSF, POTS, MCAS, or SFN. The majority (131/191) of cases qualified simultaneously for more than one of the above syndromes. One third (65/191) of cases even qualified simultaneously for all four syndromes. In contrast, 16% (31/191) of cases could not be assigned to any one of the four syndromes (Figure 1). These observations support the previous proposition [1,2] that PACVS constitutes a disease or syndrome, sui generis, that shares certain features with, but is distinct from, ME/CSF, POTS, MCAS, or SFN. Therefore, we readdressed the data with an unbiased approach.

### 3.2. Unbiased Clustering of PACVS-Associated Symptoms

**Figure 2:**
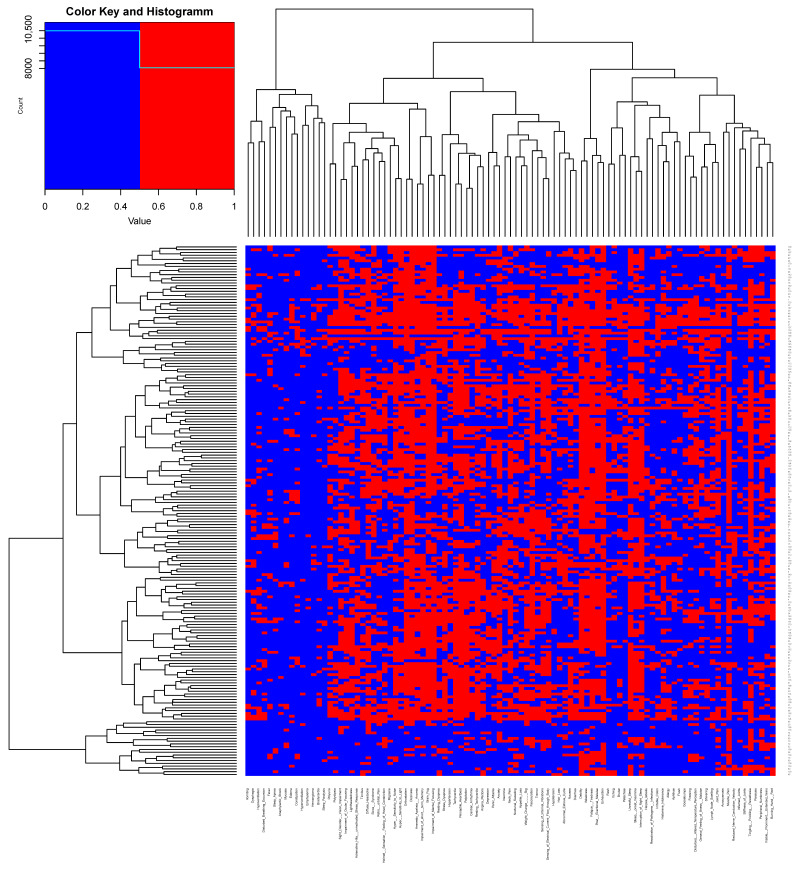
Hypothesis-free clustering of PACVS-associated symptoms. Hierarchical clustering of symptoms reported by the participants of the PACVS study cohort was performed using a modified Jaccard index considering distances of similarities and dissimilarities. The eight clusters indicated at the bottom were selected at the level h = 1.8 based on medical knowledge using the cut-tree function of the computer language R (details of clusters are in Table 2).

To delineate the clinical characteristics of *PACVS* in a hypothesis-free manner, we performed cluster analyses of the symptoms and diagnoses listed in Table 1. The clearest results were obtained by analyzing Jaccard distances of similarities and dissimilarities (Figure 2). Eight major clusters of symptoms were thus identified (Figure 2, bottom). These clusters appeared plausible and meaningful in medical terms since they exhibited a reasonable degree of coherence and were consistent with established etiologies as follows: cluster 1 comprised symptoms of generally reduced well-being commonly associated with serious consuming diseases (B-symptoms); cluster 2 encompassed symptoms of peripheral nervous dysfunction; cluster 3 contained the core symptoms of chronic fatigue and malaise; cluster 4 contained key symptoms of cardiovascular dysfunction; cluster 5 comprised common migraine-like symptoms (cognitive impairment, headache, visual, and acoustic dysfunction); cluster 6 encompassed symptoms of psychomotor disturbances and anxiety disorder; cluster 7 combined sleep disturbances and cutaneous symptoms; and the symptoms assembled in cluster 8 were consistent with disturbed autonomous regulation of breathing and eating. Table 2 provides a complete listing of the symptoms in each of the above symptom clusters.

### 3.3. Prevalence of Major PACVS-Associated Symptoms

**Figure 3:**
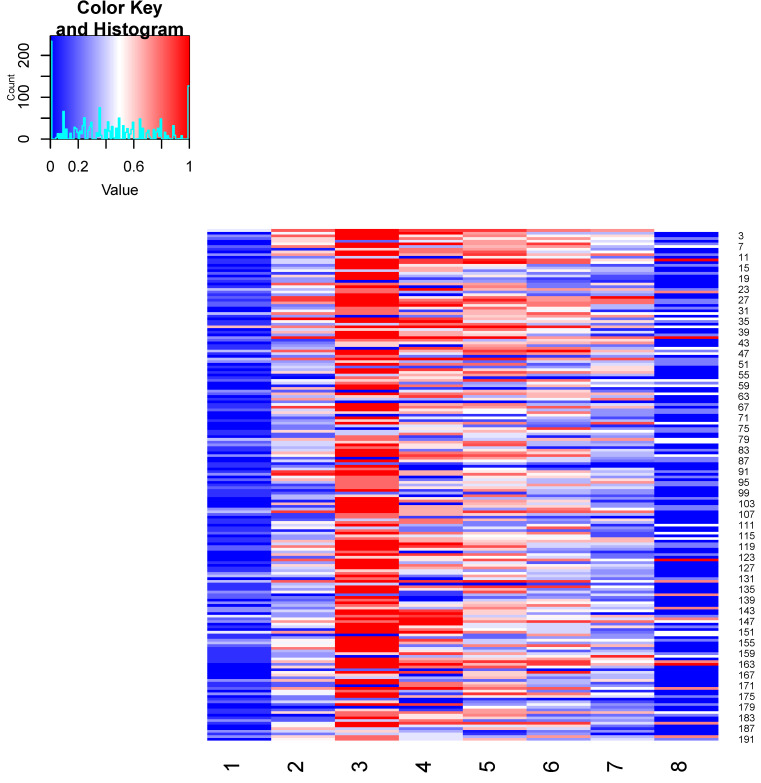
Matching of study participants to symptom clusters. Study participants were assigned to the eight clusters (Figure 2) based on the modified Jaccard index (mean of Jaccard index and Jaccard index of the negated values). Participants highlighted in red strongly matched the symptom cluster, those in white moderately matched the cluster, and those in blue matched the cluster poorly or not at all (value = 1: strong correlation with the cluster based on the modified Jaccard index; value = 0: no correlation with the cluster based on the modified Jaccard index).

Study participants were assigned to symptom clusters according to (i) proportion of symptoms in the cluster, (ii) the Jaccard index, or (iii) the modified Jaccard index (i.e., the average of the Jaccard index and the Jaccard index of the negated values) (Figure 3). The outcome of these three approaches was comparable: almost all participants fit into cluster 3 (chronic fatigue, malaise), which apparently comprises a cross-cohort symptomatology characteristic of PACVS. Cluster 2 (peripheral neuropathy, dysesthesia, motor weakness, pain, vasomotor dysfunction), cluster 4 (cardiovascular impairment), and cluster 5 (cognitive impairment, headache, visual and acoustic dysfunctions) were also frequently represented in the study cohort, and mostly overlapping with the symptoms of cluster 3. The set of symptoms represented by these clusters apparently characterizes subgroups of PACVS symptomatology. Of note, by the Jaccard index alone, more patients were assigned to cluster 5 than to cluster 3. In contrast, almost no participants fit into cluster 1 (general unwellness) or cluster 8 (dysphagia, breathing impairment), which appear to comprise nonspecific symptoms not characteristic of PACVS.

### 3.4. Abnormalities of Blood Markers in PACVS

We have recently reported that PACVS can be discriminated from the normal vaccination response by alterations in certain receptor antibodies (increases in angiotensin II type 1 receptor antibodies and decreases in alpha-2B adrenergic receptor antibodies) and increases in IL-6 and IL-8 [2]. In addition, here, we investigated the sera of the study participants using an extended panel of established blood markers for organ-specific dysfunctions. The value distribution in the study cohort was compared to z-scores (sNFL) or age-adjusted normal ranges of these parameters in healthy individuals.

As summarized in Table 3, the following abnormalities were observed: 17% of the cases exhibited abnormally high eGFR (>120 mL/min) combined with abnormally low serum urea (<18 mg/dL); fT3 values were in all cases either below the 95% confidence limit (34% of cases) or in the lower half of the normal range (66% of cases); 61% of cases exhibited a low ferritin-index and subnormal levels of transferrin, soluble transferrin receptor, and transferrin saturation; and 27% of cases exhibited serum levels of sNFL above the 90th percentile [12]. Almost half of the study participants exhibited IgG subclass imbalances, most notably decreased IgG-2 (44% of cases) or increased IgG-3 (11% of cases). IL-6 was increased in 60% of cases. IL-8 was increased in 90% of cases, assuming the free fraction was determined [2], and in 83% of cases assuming other factors, included that the erythrocyte-bound fraction (estimated at 80% according to [13]) was possibly released during transport of uncentrifuged serum samples (i.e., total IL-8). One third of cases even presented IL-8 values above 30,000 pg/mL, corresponding to >100-fold increases in total IL-8. Total cholesterol and triglycerides were increased in 30 and 20%, respectively, of the cases. However, LDL levels and LDL/HDL ratios were not increased accordingly, and fasting before serum sampling was not assured. Cardiac markers and parameters of liver function exhibited a normal distribution in the study cohort.

Associations of abnormally distributed blood markers with symptom clusters (Table 2) were undetectable or insignificant (*p* > 0.0001). Symptoms/symptom clusters were also not correlated with altered receptor antibodies [2], nor with COVID-19 reconvalescence (indicated in the case history and/or by serological NAB reactivity), nor with any particular SARS-CoV-2 vaccination regimen [2], nor with the state of SARS-CoV-2 virus immunization indicated by SAB reactivity [11]. Receptor antibodies, COVID-19 reconvalescence, and SAB-reactivity were also not significantly correlated/associated with the abnormalities of routine laboratory markers listed in Table 3.

## 4. Discussion

### 4.1. Comparison of the Clinical Phenotypes of PACVS and ME/CFS

The vast majority of cases included in this study [2] exhibited in varying proportions a symptomatic triplet of (i) malaise/chronic fatigue, (ii) cognitive impairment, and (iii) peripheral neuropathic symptoms. Consequently, the majority of participants conformed to ME/CFS. However, a large subgroup (131/191) also fit several other established complex dysautonomia syndromes, whereas a substantial minor subgroup (31/191) fit none of these. Given the leading symptoms of chronic fatigue and malaise, ME/CFS seemed to be the best fit. Thus, PACVS could be a sub-form of ME/CFS, a notion possibly to be corroborated by the inclusion of more or other diagnostic criteria and/or the application of different cut-off points concerning disease severity. On the other hand, the ambiguity of assignment of PACVS-affected individuals to a variety of established complex dysautonomia syndromes could indicate that PACVS is a syndrome sui generis sharing some but not all features with these syndromes.

### 4.2. Comparison with Other Sequelae of SARS-CoV-2 Vaccination

Long-termed sequelae following SARS-CoV-2 vaccination in published case reports encompass dysfunctions of the eyes (scintillating scotoma, double vision), malaise (fever, myalgia, fatigue), renal dysfunction (nephrotic syndrome, IgA nephropathy, lupus nephritis), and a variety of other auto-immune manifestations [15,16,17]. Apart from malaise, none of these sporadic sequelae of SARS-CoV-2 vaccination figured prominently among the clinical features reported by the majority of the participants of this study. Most notably, Guillain-Barré syndrome (GBS), observed in association with SARS-CoV-2 vaccination in more than 100 cases worldwide [18,19], was not presented by a single one of the 191 cases studied here, even though GBS and associated symptoms were explicitly addressed in the recruitment questionnaire of our study. Interestingly, the association of GBS with SARS-CoV-2 vaccination has been attributed to the reactivation or exacerbation of pre-existing GBS [20]. In analogy, many other sequelae occasionally reported in the context of SARS-CoV-2 vaccination may similarly be based on the exacerbation or reactivation of preexisting or dormant diseases. In contrast, the panoply of symptoms reported by the participants of this study seems to occur de novo and to share similarities with endogenous complex dysautonomia syndromes such as ME/CFS, MCAS, POTS, and SFN.

### 4.3. PACVS-Associated Alterations of Diagnostic Markers

We have recently demonstrated [2] that PACVS can be discriminated from the normal post-vaccination state by increases in interleukins 6 and 8 and alterations of certain receptor antibodies. Here we demonstrate a variety of further blood markers, alterations of which are associated with PACVS. These alterations may add to the general characterization of PACVS as a disease entity, but they were not significantly associated with specific subsets of symptoms presented by the study participants. The following notable alterations of blood markers were observed in the participants of our study.

#### 4.3.1. Increased eGFR

Abnormally high eGFR combined with abnormally low serum urea, as presented by 17% of the study cases, could possibly indicate insipid diabetes/hypopituitarism, which has occasionally been observed in conjunction with SARS-CoV-2 mRNA vaccination [21,22,23]. In keeping with that notion, a third of the study participants reported clinical symptoms fitting hypopituitarism, e.g., diabetes insipidus (i.e., polyuria, polydipsia and hypotonia, see: Table 1). However, these symptoms were not correlated with abnormally high eGFR or abnormally low serum urea. Regarding the thyroid axis, we did not observe abnormally low TSH levels. However, we neither measured copeptin nor performed any functional endocrinological testing.

#### 4.3.2. Low fT3

More than 90% of the study participants presented unimpaired thyroid function based on reference values of TSH and fT4. Abnormalities of TSH and fT4 exhibited by <10% of the cases could be mainly related to pre-existing auto-immune thyroid diseases reported in the case history and/or indicated by increases in TPO antibodies (18 cases) or TSH-R antibodies (1 case). All study participants exhibited low fT3 values, which in conjunction with normal fT4 and normal TSH are thought to reflect oblique or incipient hypothyroidism as a nonspecific epiphenomenon of critical illness, also addressed as “low-T3-syndrome” [24]. In keeping with that notion, fT3 levels did not exhibit a different distribution in the eight symptom clusters, which were indistinguishable from each other based on fT3 values alone.

#### 4.3.3. Disturbance of Iron Storage

In this study, 61% of PACVS subjects exhibited a low ferritin index and subnormal levels of transferrin, soluble transferrin receptor, and transferrin saturation. These abnormalities were not correlated with age or gender, suggesting enhanced iron loss due to menstrual bleeding an unlikely cause. Imbalances in iron metabolism and iron storage as suggested by the above markers are a possible cause of chronic fatigue reported by a majority of study participants. However, the above abnormalities were not significantly correlated to chronic fatigue reported as a single symptom, nor was the distribution of these values significantly different between the eight symptom clusters (*p* > 0.0001). Of note, the indications of altered iron metabolism and decreased iron storage observed here in the context of PACVS did not encompass hyper-ferritinemia, which is frequently observed in severe cases [25] or chronic sequelae [26] of COVID-19.

#### 4.3.4. Increase in Serum Neurofilament Light Chain (sNFL)

Here, 27% of the PACVS cohort exhibited serum levels of the neuroaxonal damage marker sNFL above the 90th percentile [12]. However, increased sNFL was not significantly associated with symptoms or symptom clusters of central or peripheral neurological dysfunction. It must be taken into account, however, that possible neuronal dysfunctions reported by the study participants were not corroborated by a standardized neurological examination, nor by electrophysiological measurements of nerve conductivity or nuclear magnetic resonance imaging of the central nervous system.

#### 4.3.5. IgG Subclass Imbalances

Almost half of the study cohort exhibited IgG subclass imbalances, most notably decreases of IgG-3 and/or increases in IgG-4. Decreases in IgG-3 have also been observed in ME/CFS unrelated to vaccination [27,28], influenza [29] and long COVID/PACS [30]. In long COVID/PACS, IgG-3 decreases are thought to reflect alterations in spike S1 protein-specific IgG subclass utilization [30,31]. The same may hold true for IgG-3 decreases here observed in PACVS. The phenomenon is of potential disease relevance, since IgG-3 is considered a protective factor against long COVID/PACS and ME/CFS [32]. However, IgG-3 decreases here observed in PACVS were not associated with specific symptoms. Moreover, PACVS-associated symptom clusters were not distinguished by the levels of IgG subclasses.

#### 4.3.6. Systemic Inflammation

A diagnostic feature presented by almost all the study participants was a significant increase in IL-6 and/or IL-8 in combination with normal CRP. This constellation distinguishes PACVS cases from the normal healthy post-vaccinations state [2]. Increased blood levels of IL-6 and IL-8 are considered early and sensitive albeit nonspecific markers of increased and persistent systemic inflammation. Most notably, increases in total IL-8 observed in ME/CFS occurring after COVID-19 are thought to support the notion that a state of persistent systemic hyperinflammation is one cause for prolonged or more severe courses of that disease [33]. It is conceivable that chronically enhanced systemic inflammation may also be the cause of low fT3 levels and alterations of iron metabolism observed in a significant fraction of the participants of this study. Moreover, persistent systemic inflammation would be a plausible cause of malaise, chronic fatigue, and other symptoms frequently reported by the study participants (see Table 2, clusters 2, 3, and 5).

#### 4.3.7. Lipids

In view of the age distribution of the study participants, the significant increases in cholesterol and triglycerides observed in one third of the cohort would be noteworthy. However, these increases were not matched by corresponding increases in LDL or the LDL/HDL ratio. Therefore, they probably do not reflect a genuine disorder of lipid metabolism. More likely they are due to a non-fasting state during sample acquisition.

### 4.4. Pathogen Reactivation

Prolonged courses and chronic sequelae of COVID-19 (long COVID/PACS [1]) are associated with reactivation of pathogenic viruses, most notably herpes virus types 6 and 7, herpes simplex virus, Epstein-Barr-virus, varicella-zoster virus, and hepatitis-B/C virus. Pathogen activation is considered a potential cause of PACS-associated symptoms overlapping with ME/CSF and other complex dysautonomia syndromes [34]. Among the PACVS-afflicted individuals studied here, pathogen activation was reported with a prevalence of 26.7% (Table 1). It was associated with sleep disturbance and cutaneous symptoms, including herpes labialis (cluster 7). These symptoms made only a minor contribution to the overall clinical presentation of PACVS (see Table 2 and Figure 3). Thus, pathogen reactivation appears to occur in PACS as well as PACVS. However, in PACS it is thought to play a major pathogenic role and is clinically associated with chronic fatigue, whereas in PACVS it seems to contribute only insignificantly to the clinical manifestations and is not associated with chronic fatigue.

## 5. Limitations

Any conclusions to be drawn from our study must take into account that the proposed disease phenotype of PACVS is solely based on symptoms, afflictions, and ailments reported by the study participants or their local doctors. These reports were not corroborated by neurological examinations, objective measurements of nerve function, imaging of nerve structures, or other clinical investigations performed in the context of the study in a stratified and standardized manner. Moreover, the pre-analytic conditions of laboratory analyses performed on blood samples of the study participants were insufficiently controlled. In particular, the time lapse between drawing blood and separation of serum was comparatively long (up to 48 h at 4 °C), and we cannot exclude that some of the laboratory results were thereby affected. Most notably, we are unable to ascertain whether free or total IL-8 was determined. However, this distinction does not alter the overall result regarding the prevalence of systemic hyperinflammation in the study cohort.

## 6. Conclusions

The data presented here and in our previous publication [2] outline a chronic syndrome probably triggered by SARS-CoV-2 mRNA vaccination. This putative syndrome, commonly addressed as PACVS [1], is probably not the only chronic health impairment associated with SARS-CoV-2 vaccination. It must be distinguished from the (re-)activation of GBS [18,19,20] and other immunologic diseases [15,16,17], which have also been observed in the context of SARS-CoV-2 vaccination. The clinical presentation of PACVS based on a study registry comprises a symptomatic triplet of (i) malaise/chronic fatigue (symptom cluster 3), (ii) migraine-like symptoms/cognitive impairment (symptom cluster 5), and (iii) peripheral nerve dysfunction (symptom cluster 2). Based on these symptoms, PACVS is either a sub-form of ME/CFS or a novel syndrome sui generis that is close to ME/CFS. Apart from the clinical presentation, PACVS may be characterized (and distinguished from the normal post-vaccination state) by a characteristic set of blood markers: almost all PACVS cases studied here exhibited increases in interleukins 6 and 8, alterations of certain receptor antibodies [2], and low fT3 levels [24]. More than half of the cases exhibited imbalances in IgG subclass distributions and in serological indications of impaired iron storage. A smaller subset of cases presented markers of renal hyper-perfusion (17%) or significant increases in sNFL (30%). However, none of these laboratory findings were limited to clinical subsets, nor were the above biomarkers linked to any specific symptom constellation of PACVS as derived from Jaccard clustering.

## Figures and Tables

**Table 1:** Prevalence of symptoms and diagnoses in PACVS study cohort.

Symptom/Diagnosis ^1^	Prevalence (%)	Syndrome Assoc. ^2,3^
Exhaustion	84.82	ME/CFS, POTS
Debility	83.77	ME/CFS, POTS
Muscle Pain	80.63	ME/CFS, POTS, SFN
Unrestful Sleep	80.63	ME/CFS
Dizziness	80.10	ME/CFS, POTS, SFN
Tingling/Prickling/Paresthesia	79.58	SFN
Impairment of Mental Focusing	78.53	ME/CFS, POTS
Fatigue/Tiredness	76.96	ME/CFS, POTS
Orthostatism	76.44	ME/CFS, POTS, SFN
Brain Fog	76.44	ME/CFS, POTS
Interruption of Night Sleep	74.87	ME/CFS, POTS
Weakness	74.35	ME/CFS, POTS
Perceptible Heartbeat	72.77	ME/CFS, POTS, SFN
Post-Exertional Malaise	70.68	ME/CFS
Fasciculation	70.68	ME/CFS, POTS, SFN
Anxiety	69.11	ME/CFS, POTS
Injury from Immunization	67.54	
Tachycardia	65.97	ME/CFS, POST, SFN
Impairment of Short-term Memory	65.45	ME/CFS, POTS
Hyper-Sensitivity to Noise	64.92	ME/CFS, POTS
Sleep-onset Insomnia	64.40	ME/CFS, POTS
Neck Pain	64.40	
Diffuse Headache	63.35	ME/CFS, POTS, MCAS
Peripheral Numbness	62.83	SFN
Amnestic Aphasia/Anomia	60.73	ME/CFS, POTS
Joint Pain	60.73	ME/CFS
Sight Disorder/Vision Impairment	60.21	ME/CFS
Stress Dyspnea	59.69	ME/CFS, MCAS
Palpitation	58.64	ME/CFS, POTS, SFN
Sensing of Internal Vibrations	58.12	SFN
Lightheadedness	57.59	ME/CFS, POTS
Resting Tachycardia	57.07	ME/CFS, POTS, SFN
Sensing Electr. Current Flow through Body	56.02	SFN
Impairment of Ocular Focusing	56.02	ME/CFS
Hyper-Sensitivity to Light	55.50	ME/CFS, POTS
Weight Change ≥/≤ 5 kg	53.93	ME/CFS, POTS
Nausea	52.36	ME/CFS, POTS, MCAS
Sicca Syndrome	49.74	ME/CFS, SFN
Tinnitus	48.17	
Post-Vaccine Syndrome	47.64	
Nocturnal Sweating	47.12	
Angina Pectoris	46.40	
Burning Hand/Feet	46.07	SFN
Histamine Intolerance	44.50	ME/CFS
Retro-orbital Pain	43.98	ME/CFS, POTS
Appetite Loss	43.46	
Panic Attacks	43.46	
Cardiac Arrhythmia	42.93	ME/CFS, POTS, SFN
Abnormal Estrous Cycle	42.93	
Disturbed/Altered Temp Perception	40.84	SFN
Freezing	40.31	
Diarrhea	40.31	POTS, MCAS
General Feeling of Illness/Malaise	39.27	ME/CFS
Resting Dyspnea	39.27	MCAS
Adrenal Hits/Unmotiv. Stress-Response	39.27	
Helmet Sensation/Head Constriction	39.27	ME/CFS, POTS, MCAS
Lymph Node Swelling	38.74	ME/CFS
Visible/Prominent/Extended Veins	37.70	
Polyuria	37.17	ME/CFS, POTS, SFN
Hair Loss/Alopecia	37.17	
Insomnia	36.13	ME/CFS, POTS
Long COVID without COVID Infection	36.13	
Hypertension	34.55	
Tremor	34.55	SFN
Depression	32.98	ME/CFS
Shivering	32.46	
Polydipsia	31.94	
ME/CFS	31.94	
Paralysis	31.41	SFN
Migraine	30.37	ME/CFS, POTS
Allergy	29.84	ME/CFS
Stiffness of Joints	29.32	
Itching	29.32	MCAS
Irritable Colon	28.27	ME/CFS
Spont. Bruising (unrelated to trauma)	27.75	
Reactivation of Pathogens/Infections	26.70	
Hypotension	26.18	ME/CFS, SFN, MCAS
Rash	26.18	POTS
Aphthae	24.61	
Goosebumps	24.08	
Vaccine-Induced Inflamm. Response	24.08	
Petechiae	23.04	
Hypoventilation	21.99	
Hyper-Inflammation	21.99	POTS
Herpes labialis	21.47	
POTS	20.94	
Inflamed Joints	20.42	
Flush	20.42	MCAS
Fever	19.90	
Acrocyanosis	19.90	POTS
Reduced Nerve Conduction Velocity	19.90	POTS, MCAS
Dysphagia	19.90	POTS
MCAS	19.37	
Myocarditis/Pericarditis	18.85	
Fibromyalgia	17.80	
SFN	17.80	
Constipation	17.28	
Edema	14.14	MCAS
Bradycardia	13.61	POTS, SFN
Disturbed Breathing Excursion	13.61	
Mycosis	12.57	
Vomiting	12.57	
Sleep Apnea	12.04	MCAS
Sleep Paralysis	7.85	
Hyperventilation	7.85	
Hemangioma	7.33	POTS
Thrombosis	6.81	
Anaphylactic Shock	5.76	MCAS
Shingles	5.76	
Guillain-Barré Syndrome	4.71	
Emphysema	3.66	
Facial Nerve Paresis	3.14	

^1^ As self-reported and/or determined by general practitioner; quartiles separated by blanks. ^2^ ME/CFS—myalgic encephalomyelitis/chronic fatigue syndrome, POTS—postural orthostatic syndrome, MCAS—mast cell activation syndrome, SFN—small fiber neuropathy. ^3^ Definition of syndrome association according to [4,5,7,8].

**Table 2:** PACVS-associated symptoms clustered according to Jaccard distances ^1^.

Cluster 1: General Unwellness • Fever (38) • Constipation (33) • Edema (27) • Bradycardia (26) • Mycosis (24) • Sleep Apnea (23) • Sleep Paralysis (15) • Hyperventilation (15) • Hemangioma (14) • Anaphylactic Shock (11) • Emphysema (7)
Cluster 2: Peripheral Nerve Dysfunction, Dysesthesia, Paralysis, Pain, Vasomotor Dysfunction • Muscle Pain (154) • Tingling or Prickling Paresthesia (152) • Peripheral Numbness (120) • Joint Pain (116) • Burning Hands or Feet (88) • Disturbed Altered Temperature Perception (78) • Freezing (77) • General Feeling of Illness/Malaise (75) • Lymph Node Swelling (74) • Visible Prominent Extended Veins (72) • Shivering (62) • Paralysis (60) • Stiffness of Joints (56) • Goosebumps (46) • Inflamed Joints (39) • Acrocyanosis (38) • Reduced Nerve Conduction Velocity (38)
Cluster 3: Chronic Fatigue, Malaise • Exhaustion (162) • Debility (160) • Fatigue, Tiredness (147) • Weakness (142) • Post-Exertional Malaise (135)
Cluster 4: Cardiovascular Impairment • Perceptible Heartbeat (139) • Tachycardia (126) • Stress Dyspnea (114) • Palpitation (112) • Resting Tachycardia (109) • Angina Pectoris (89) • Cardiac Arrhythmia (82) • Resting Dyspnea (75) • Hypertension (66)
Cluster 5: Cognitive Impairment, Headache, visual and acoustic Dysfunctions • Dizziness (153) • Impairment of Mental Focusing (150) • Orthostatism (146) • Brain Fog (146) • Impairment of Short-term Memory (125) • Hyper-Sensitivity to Noise (124) • Diffuse Headache (121) • Amnestic Aphasia, Anosmia (116) • Sight Disorder, Vision Impairment (115) • Lightheadedness (110) • Impairment of Ocular Focusing (107) • Hyper-Sensitivity to Light (105) • Sicca Syndrome (95) • Tinnitus (92) • Retro-orbital Pain (84) • Adrenaline Hits, Unmotivated Stress Response (75) • Helmet Sensation or Feeling of Head Constriction (75) • Polyuria (71) • Polydipsia (61) • Migraine (59)
Cluster 6: Psychomotoric Dysfunction, Anxiety, Disturbed Body Perception, Gastrointestinal Dysfunction • Fasciculation (135) • Anxiety (132) • Neck Pain (123) • Sensing of Internal Vibrations (111) • Sensing of Electrical Current Flow through Body (107) • Weight Change of 5 kg (103) • Nausea (100) • Nocturnal Sweating (90) • Appetite Loss (83) • Panic Attacks (83) • Abnormal Estrous Cycle (82) • Diarrhea (77) • Hair Loss (71) • Tremor (66) • Depression (63) • Irritable Colon (54) • Hypotension (50)
Cluster 7: Sleep Disturbance, Cutaneous Symptoms • Unrestful Sleep (154) • Interruption of Night Sleep (143) • Sleep-onset Insomnia (123) • Insomnia (69) • Histamine Intolerance (85) • Allergy (57) • Itching (56) • Bruises (53) • Reactivation of Pathogen Infections (51) • Rash (50) • Aphthae (47) • Petechiae (44) • Herpes labialis (41) • Flush (39)
Cluster 8: Dyspagia, Breathing Impairment • Dysphagia (38) • Hypoventilation (42) • Disturbed Breathing Excursion (26) • Vomiting (24)

^1^ Clustering detailed in Figure 2; number of affected participants given in brackets.

**Table 3:** Abnormalities of serum markers in PACVS study cohort.

	Normal Range (n.r.) ^1^ [Dimension]	Below n.r. ^2^ *n* (%)	Above n.r. ^2^ *n* (%)	Remarks
Kidney				GFR > 120 always associated with urea <18
eGFR (CAPA)	60–120 [mL/min/1.73 m^2^]	14 (7)	33 (17)	
eGFR (CKD-EPI)	60–120 [mL/min/1.73 m^2^]	1 (1)	30 (16)	
Urea	♀ 21–43, ♂ 18–55 [mg/dL]	70 (37)	1 (1)	
Thyroid				All other fT3-values in lower half of n.r.
TSH	0.27–4.2 [µIU/mL]	4 (2)	4 (2)	
fT3	♀ 2.6–5, ♂ 2–4.4 [ng/mL]	65 (34)	-	
fT4	♀ 9.8–16.3, ♂ 9.3–17.1 [pg/mL]	14 (7)	7 (4)	
anti-TPO	≤34 [IU/mL)	-	18 (9)	
anti-TSH-R	≤1.7 [IU/L]	-	1 (1)	
Cardiac markers				
ProBNP	≤125 [pg/mL]		-	
hsTroponin T	♀ ≤ 14, ♂ ≤ 11 [ng/L]		1 (1)	
Liver				AST/ALT ≤ 1 in all cases No value > 2
AST	♀ ≤ 3, ♂ ≤ 35 [U/L]		20 (10)	
ALT	♀ ≤ 35, ♂ ≤ 45 [U/L]		11 (6)	
ALP	♀ ≤ 104, ♂ ≤ 129 [U/L]		1 (1)	
gammaGT	♀ ≤ 38, ♂ ≤ 55 [U/L]		7 (4)	
Bilirubin	≤1.2 [mg/dL]		8 (4)	
Lipids				Fasting state not assured
Cholesterol	≤200 [mg/dL]		61 (32)	
Trigliceride	≤150 [mg/dL]		39 (20)	
LDL	≤160 [mg/dL]		10 (5)	
HDL	≥40 [mg/dL]	32 (17)		
LDL/HDL	2.5–3.5	117 (62)	4 (2)	
Inflammation				IL-6 up to 50,000 IL-8 up to 2,000,000
Il-6	≤7 [pg/mL]	-	115 (60)	
Il-8	≤62 [pg/mL]	-	172 (90)	
CRP	≤0.5 [mg/dL]	-	13 (7)	
IgG subclasses				
total	700–1600 [mg/dL]	19 (10)	3 (2)	
IgG1	280–800 [mg/dL]	1 (1)	18 (9)	
IgG2	115–570 [mg/dL]	2 (1)	5 (3)	
IgG3	24–125 [mg/dL]	88 (46)	17 (9)	
IgG4	5.2–125 [mg/dL]	12 (6)	21 (11)	
Iron metabolism				
Fe	♀ 37–165, ♂ 40–155 [µg/dL]	9 (5)	20 (10)	
Ferritin	♀ 13–150, ♂ 30–400 [µg/L]	11 (6)	3 (2)	
Transferrin	200–360 [mg/dL]	34 (18)	3 (2)	
Transf. Saturation	16–45 [%]	27 (14)	20 (10)	
Sol Transf. Recept.	0.81–1.75 [mg/L]	36 (19)	4 (2)	
Ferritin Index	0.63–2.2	117 (61)	2 (1)	
Neurodegeneration				Adjusted for age, BMI, GFR according to ref. [12]
sNFL	<1.5 (zScore)		39 (20)	
sNFL	10–90 (Percentile)	6 (3)	52 (27)	
BMI	18.5–30	19 (10)	16 (8)	

^1^ Following IFCC unless stated otherwise. ^2^ Figures in red indicate that >10% of the values were above or below normal ranges.

## Data Availability

The data presented in this study are available on request from the corresponding author. The data are not publicly available due to privacy concerns by the study participants.

## Abbreviations

BMI	Body mass index
CRP	C-reactive protein
eGFR	Estimate of glomerular filtration rate based on serum creatinine or cystatin C
fT3	Free tri-iodine thyroxine
fT4	Free tetra-iodine thyroxine
GBS	Guillain-Barré syndrome
HDL	High-density lipoptotein
LDL	Low-density lipoptotein
MCAS	Mast cell activation syndrome
ME/CFS	*Myalgic encephalomyelitis*/chronic fatigue syndrome
NAB	PanIg reactivity against SARS-CoV-1 nucleocapsid protein
panIg	Immunoglobulins of all classes (A, D, G, E, M)
pBNP	Pro-brain natriuretic peptide
PEM	Post exertional malaise
POTS	Postural tachycardia syndrome
PACS	Post-acute COVID-19 syndrome
PACVS	Post-acute COVID-19 vaccination syndrome
ROC	Receiver-operator characteristics
SAB	PanIg reactivity against SARS-CoV-2 spike S1 protein
SFN	Small fiber neuropathy
sNFL	Serum neurofilament light chain
TPO	Thyroid peroxidase
TSH	Thyroid-stimulating hormone
TSH-R	Receptor for thyroid-stimulating hormone
